# Manual muscle strength testing of critically ill patients: feasibility and interobserver agreement

**DOI:** 10.1186/cc10005

**Published:** 2011-01-28

**Authors:** Catherine L Hough, Binh K Lieu, Ellen S Caldwell

**Affiliations:** 1 Division of Pulmonary and Critical Care Medicine, Department of Medicine, University of Washington, 325 Ninth Avenue, Mailstop 359762, Seattle, WA 98104, USA

## Abstract

**Introduction:**

It has been proposed that intensive care unit (ICU)-acquired weakness (ICUAW) should be assessed using the sum of manual muscle strength test scores in 12 muscle groups (the sum score). This approach has been tested in patients with Guillain-Barré syndrome, yet little is known about the feasibility or test characteristics in other critically ill patients. We studied the feasibility and interobserver agreement of this sum score in a mixed cohort of critically ill and injured patients.

**Methods:**

We enrolled patients requiring more than 3 days of mechanical ventilation. Two observers performed systematic strength assessments of each patient. The primary outcome measure was interobserver agreement of weakness as a binary outcome (ICUAW is sum score less than 48; "no ICUAW" is a sum score greater than or equal to 48) using the Cohen's kappa statistic.

**Results:**

We identified 135 patients who met the inclusion criteria. Most were precluded from study participation by altered mental status or polytrauma. Thirty-four participants were enrolled, and 30 of these individuals completed assessments conducted by both observers. Six met the criteria for ICUAW recorded by at least one observer. The observers agreed on the diagnosis of ICUAW for 93% of participants (Cohen's kappa = 0.76; 95% confidence interval (CI), 0.44 to 1.0). Observer agreement was fair in the ICU (Cohen's kappa = 0.38), and agreement was perfect after ICU discharge (Cohen's kappa = 1.0). Absolute values of sum scores were similar between observers (intraclass correlation coefficient 0.83; 95% CI, 0.67 to 0.91), but they differed between observers by six points or more for 23% of the participants.

**Conclusions:**

Manual muscle testing (MMT) during critical illness was not possible for most patients because of coma, delirium and/or injury. Among patients who were able to participate in testing, we found that interobserver agreement regarding ICUAW was good, particularly when evaluated after ICU discharge. MMT is insufficient for early detection of ICU-acquired neuromuscular dysfunction in most patients and may be unreliable during critical illness.

## Introduction

Patients with acute respiratory failure, shock and other manifestations of critical illness or injury are at risk of developing neuromuscular dysfunction as a result of entities such as critical illness polyneuropathy, critical illness myopathy and disuse atrophy [1-3]. Many of these patients have severe weakness which is detectable on the basis of a clinical strength evaluation. This severe weakness has been termed "intensive care unit-acquired paresis" [4] or ICU-acquired weakness (ICUAW) [5]. This condition is diagnosed on the basis of the average Medical Research Council (MRC) strength score combined for 12 specified muscle groups (the MRC sum score) being less than 48, indicating that average strength is limited to movement against gravity and partial resistance [6]. Observational studies have reported that ICUAW is common, with an incidence of 25% [4,7], and is associated with poor outcomes, including mortality [7], prolonged mechanical ventilation [4,7,8] and the need for additional institutional care after hospital discharge [7].

Systematic strength assessment and the definition of ICUAW according to the MRC sum score is becoming more common in research [9] and has been recommended for both research and clinical practice [5]. However, little is known about the feasibility or test characteristics of manual muscle testing (MMT) or about ICUAW as a dichotomous diagnosis on the basis of the MRC sum score for the general population of patients with critical illness. There are two groups for whom studies of interobserver agreement of MRC sum scores have been described: patients with Guillain-Barré syndrome [6] and ICU survivors after hospital discharge [10]. Compared with these groups, ICU general population patients are less likely to be able to cooperate with volitional strength assessment and more likely to have limited access to their extremities because of trauma, burns and treatment involving medical devices. We sought to determine the feasibility of assessment and interobserver agreement regarding the diagnosis of ICUAW and the MRC sum score in a mixed cohort of critically ill and injured patients.

## Materials and methods

### Study design

We conducted a prospective study of critically ill patients at a single academic county hospital in Seattle, WA, USA, during 4 months in 2006 and 2007. We obtained approval from the institutional review board of the University of Washington for all study procedures.

### Screening and eligibility

Critically ill patients were consecutively screened for eligibility after 48 hours in the ICU. Inclusion criteria included age at least 18 years, at least 3 days of mechanical ventilation for acute respiratory failure and the expectation that the patient would be able to follow complex commands. We excluded patients with spinal cord injury, stroke, injury preventing the evaluation of six or more muscle groups, inability to follow complex commands during the follow-up period, inability to understand English and inability to provide informed consent.

### Study procedures

Eligible patients were screened 5 days each week for attention and comprehension on the basis of their responses to five orders as described by De Jonghe *et al. *[4]. Screening was coordinated with daily interruption of sedation according to institutional protocol. Once the patient was able to follow at least three orders consistently, two observers performed the structured MMT: BKL, a senior medical resident, and CLH, an attending critical care physician. Prior to this study, both observers completed multistep training in performance of MMT that included the creation of a detailed MMT instruction manual, didactic teaching of each other and other healthcare professionals and supervised practice in and out of the ICU setting (with a standardized patient as well as practice patients in the ICU). The order of observer assessment was random (determined by coin flip). Examinations were performed independently within 30 minutes of each other. The second observer was blinded to the results of the first observer's evaluation.

Each observer repeated the attention screen and then performed the 12 muscle group strength assessment: bilateral shoulder abduction, elbow flexion, wrist extension, hip flexion, knee extension and foot dorsiflexion. The patient was positioned in either the sitting or supine position, depending on the patient's clinical situation. Strength in each muscle group was scored according to the six-point MRC system, in which a score of 0 was no contraction, 1 was a flicker of contraction, 2 was active movement with gravity eliminated, 3 was active movement against gravity, 4 was active movement against gravity and resistance and 5 was normal power [11]. If the patient would not or could not perform the test for an individual muscle group, no score was recorded and data were indicated as missing.

### Data collection and statistical analyses

We collected demographic and hospital variables from electronic medical records. Admission diagnoses and ICU complications (the presence of ventilator-associated pneumonia, sepsis, acute respiratory distress syndrome, bacteremia, renal failure or *Clostridium difficile *colitis) were abstracted from hospital discharge summaries. We calculated statistics (means ± standard deviation (SD), medians with interquartile range (IQR) and proportion or binomial confidence interval (CI)) to describe the cohort. We calculated one MRC sum score for each observer per patient. If the patient was unable to perform a test for a muscle group, we assigned the score on the basis of the score for the contralateral joint; if unavailable, we used the score for the ipsilateral group of muscles in the same proximity (hip: shoulder, knee: elbow or wrist: foot). ICUAW was defined as a MRC sum score less than 48.

We calculated the primary outcome measure, interobserver agreement on ICUAW, on the basis of the simple Cohen's kappa statistic [12] and the 95% confidence interval [13]. The strength ratings may be unbalanced or skewed, so the prevalence and bias-adjusted Cohen's kappa statistics [14,15] are also presented. We calculated the proportion of positive (p-pos) and the proportion of negative (p-neg) agreement between observers regarding the diagnosis of ICUAW [16]. We performed a sensitivity analysis of the effect of assigning scores on the basis of the score for the contralateral joint or for the ipsilateral group of muscles by restricting our primary analysis to patients who performed the MMT with all 12 muscle groups. We then stratified the cohort by the location of the examination (ICU versus hospital ward) and calculated the agreement in each group. Agreement was graded on a scale of "poor" to "almost perfect" as suggested by Landis and Koch [17]. Using the methodology described by Kleyweg *et al. *[6], we calculated the proportion of patients for whom the observers' sum scores differed by 10% or more (more than six points). Since the two raters were considered a random sample of the population of possible raters, intraclass correlation coefficients were computed on the basis of two-way random effects models for absolute agreement [15,18]. We also looked at the absolute agreement for each individual muscle group with the weighted Cohen's kappa statistic, comparing each observer's MRC score as an ordered categorical variable (scores from 0 to 5). Statistical calculations were performed using Stata version 10.0 software (College Station, TX, USA) and SAS version 9.2 software (SAS Institute Inc., Cary, NC, USA).

## Results

**Figure 1:**
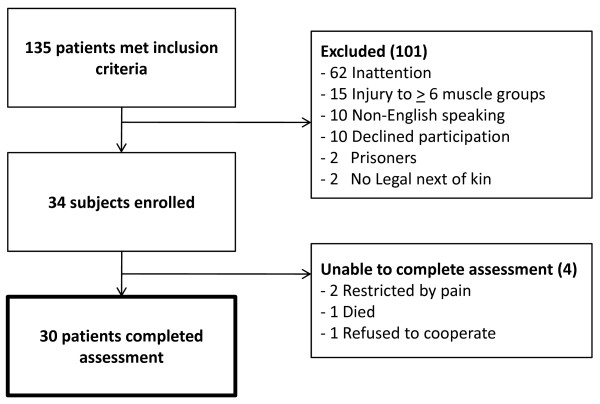
**Flowchart showing screening, enrollment and evaluation**.

A flowchart of patient screening and enrollment is presented in Figure 1. Of the 135 patients who met the inclusion criteria, nearly half did not pass the attention screen during the study period (*n *= 62; 46%). Thirty-four patients (25% of eligible patients) consented to participate in the study. These 34 patients' baseline characteristics are presented in Table 1.

**Table 1:** Patient demographics^a^

Demographic variable	Data
Age, yr (mean ± SD)	49 ± 15
Male sex, %	71%
Admission service	
Surgery, %	53%
Medical, %	32%
Neurology/neurosurgery, %	15%
Admission diagnosis^b^	
Trauma, %	44%
Infection, %	29%
Other, %	27%
Any ICU complications^c^, %	62%
Median days of mechanical ventilation prior to examination (interquartile range)	10 days (6 to 16)
Median days between eligibility and examination (interquartile range)	8 days (6 to 12)

^a^ICU, intensive care unit; ^b^"Other" admission diagnoses include chronic obstructive pulmonary disease exacerbation, congestive heart failure, variceal or subarachnoid hemorrhage, burns, drowning and alcoholic hepatitis; ^c^ICU complications include ventilator-associated pneumonia, sepsis, renal failure, bacteremia, *Clostridium difficile *colitis and acute respiratory distress syndrome.

Patients received a median of 10 days of mechanical ventilation before the study examination (IQR, 6 to 16 days). Among patients who failed to pass the attention screen, more than 1 week elapsed in most cases between the identification of an eligible patient and performance of the examination (median, 8 days; IQR, 6 to 12 days). Thirty patients were able to perform MMT administered by both observers (88%). One died before the examination, one refused to participate and two were unable to complete the assessment because of issues related to attention and pain control. Although all patients were initially identified in the ICU, only 10 examinations occurred in the ICU; the remaining 20 took place after ICU discharge to the hospital ward. Only two patients were receiving mechanical ventilation at the time of examination. Most patients who performed the MMT were tested in all muscle groups (*n *= 17; 57%). Eleven patients were tested in 10 or 11 muscle groups, and two patients were tested in seven or eight muscle groups. Table 2 presents the number of patients in whom each muscle group was assessed by both observers. We were unable to assess muscle groups most frequently because of injury, pain and the presence of medical devices (such as casts, external fixation devices and extensive burn dressings).

**Table 2:** Interobserver agreement regarding MRC score: individual muscle groups^a^

Muscle mobility	Number	Average of exams Median (IQR)	Agreement, %	**Weighted kappa (95% CI)**^**b**^	**Intraclass correlation coefficient (2, 1) (95% CI)**^**c**^
Shoulder abduction: R	28	4.5 (4 to 5)	57%	0.51 (0.32 to 0.71)	0.68 (0.43 to 0.83)
Shoulder abduction: L	27	4.5 (3.5 to 4.5)	47%	0.36 (0.12 to 0.60)	0.53 (0.21 to 0.75)
Elbow flexion: R	29	4.5 (4 to 5)	57%	0.35 (0.08 to 0.62)	0.53 (0.21 to 0.74)
Elbow flexion: L	29	4.5 (4.5 to 5)	60%	0.23 (0 to 0.55)	0.29 (0 to 0.59)
Wrist extension: R	28	5 (4.5 to 5)	80%	0.56 (0.30 to 0.82)	0.61 (0.32 to 0.79)
Wrist extension: L	30	4.5 (4.5 to 5)	73%	0.44 (0.16 to 0.73)	0.50 (0.18 to 0.72)
Hip flexion: R	26	4 (3.5 to 5)	53%	0.47 (0.25 to 0.70)	0.62 (0.33 to 0.80)
Hip flexion: L	24	4.25 (3.5 to 5)	40%	0.32 (0.11 to 0.53)	0.50 (0.17 to 0.73)
Knee extension: R	28	4.75 (4.25 to 5)	60%	0.29 (0.02 to 0.57)	0.31 (0 to 0.59)
Knee extension: L	28	4.75 (4.5 to 5)	60%	0.29 (0.02 to 0.57)	0.31 (0 to 0.59)
Foot dorsiflexion: R	26	5 (4.5 to 5)	80%	0.64 (0.43 to 0.85)	0.75 (0.54 to 0.87)
Foot dorsiflexion: L	28	5 (4.75 to 5)	40%	0.32 (0.11 to 0.53)	0.50 (0.17 to 0.73)

^a^R, right; L, left; IQR, interquartile range; CI, confidence interval; MRC, Medical Research Council; ^b^weighted kappa treating strength scale as ordinal linear weights; ^c^two-way random effects model (raters and participants treated as random effects) intraclass correlation coefficient treating strength scale as continuous.

The median MRC sum scores for each observer were 55 (IQR, 49 to 58) and 56 (IQR, 50 to 58). The continuous outcome of the MRC sum score differed by 10% or more between observers for 7 (23%) of the 30 patients. The intraclass correlation coefficient of the sum score was 0.83 (95% CI, 0.67 to 0.93). As shown in Table 2, the agreement of the scores for individual muscle groups was poor, particularly among the more proximal muscles. Agreement ranged from 40% to 83%, the weighted Cohen's kappa statistics ranged from 0.11 to 0.64 and the interclass correlation coefficients ranged from 0.15 to 0.75.

Each observer identified six patients with ICUAW (MRC sum score <48), with an incidence of 17% (95% CI, 3% to 31%). Among all patients, the interobserver agreement was 93% (Cohen's kappa = 0.76; 95% CI, 0.44 to 1.0). The prevalence and bias-adjusted Cohen's kappa was 0.87. p-pos agreement was 80%, and p-neg agreement was 96%. Assignment of missing values did not change the estimates of agreement. After stratifying the sample on the basis of the hospital location of examination, we found that both cases in which the observers disagreed occurred in the ICU. Agreement on the diagnosis of ICUAW was poor for patients examined in the ICU: Observers agreed 80% of the time (Cohen's kappa = 0.38). Agreement regarding ICUAW on the hospital ward was 100% (Cohen's kappa = 1.0). Although not statistically significant, patients evaluated in the ICU were less likely than those on the hospital ward to perform complete examinations (40% versus 65%; *P *= 0.20).

## Discussion

In our cohort of critically ill surgical and medical patients, we found that systematic MMT could not be performed in the ICU for most patients. MMT requires the attention and comprehension of the patient; thus the high prevalence of persistent coma and delirium in our population prohibited evaluation. While our institution has a sedation protocol with daily interruption of sedation, it is very likely that a more conservative least sedation [19,20] or no sedation [21] approach could have markedly decreased the number of delirious and oversedated patients and might have allowed MMT evaluation earlier in the course of the patients' critical illness. Some patients were discharged from the hospital before MMT could be performed, and others died before assessment. In other patients, extensive injury due to burns or other trauma prevented evaluation because of generalized weakness. The investigators in the two largest studies of interobserver agreement regarding MMT in critically ill patients did not investigate the ability of patients to cooperate with volitional testing [6,10]; it appears that the only patients who were included were able to fully participate in the study procedures. We expect that the issues of delirium, coma and injury would have been uncommon among both patients with Guillain-Barré syndrome patients and outpatient survivors of critical illness.

Prospective studies of ICUAW have encountered problems with the feasibility of MMT in critically ill patients. De Jonghe *et al. *[4] identified 332 critically ill medical and surgical patients who met the inclusion requirement of 7 or more days of mechanical ventilation. One hundred one patients were excluded because of neurologic disease, and 10 were excluded because of language barriers or lack of assessable limbs. Of the 206 patients remaining, more than half (*n *= 111) did not awaken often enough to be evaluated before discharge or death. It is not clear whether most strength evaluations were done in the ICU or on the ward in their study, but the authors reported that the mean delay between the onset of mechanical ventilation was 12.4 days (SD, 6.8 days). We can therefore infer that even among the minority subset of eligible patients who had been selected for their ability to cooperate with strength testing, MMT was generally not performed early in the course of their critical illness.

In a more recent study of medical ICU patients who received 5 or more days of mechanical ventilation, Ali *et al. *[7] enrolled 174 patients, and only 38 patients (22%) were unable to perform the MMT. However, 94 patients were excluded because they were "unlikely to awaken," and 40 additional patients were excluded because of inability to communicate. As such, 50% of potential patients were not included because of cognitive inability to cooperate with volitional testing. The authors did not present data about patients' location in the ICU versus the hospital ward at the time of initial evaluation. They reported that most patients were first assessed on or after the day of the cessation of mechanical ventilation (NA Ali, personal communication, April 2009). The Ali *et al. *[7] study included an assessment of interobserver agreement between two observers who examined 12 patients. They reported perfect agreement regarding the diagnosis of ICUAW but did not present the timing or location of these 12 evaluations.

In the current investigation, we found that interobserver agreement regarding ICUAW was good among patients who were able to participate in MMT, particularly in those patients evaluated after ICU discharge. The 95% CI of the Cohen's kappa statistic is wide, including both poor and almost perfect agreement, since the incidence of ICUAW was low and our sample was small. As such, we cannot be confident that interobserver agreement is as excellent as previously reported, particularly for assessments performed in the ICU. Interobserver agreement on the MRC sum score as a continuous outcome was rarely perfect and differed by 10% or more for nearly one-fourth of patients. Interobserver agreement for individual muscle groups was poor, particularly for the proximal muscles. The proximal muscles, especially the hip flexors, were the most likely not to be assessed.

This study is limited by the small sample size and the low incidence of ICUAW among the assessable patients. The strengths of this study include the diverse population of critically ill patients included and the focus on feasibility as well as the reliability of strength testing in the ICU. The intensive training in the performance the MMT protocol likely decreased the variability between observers, allowing this study to highlight the problems of attention and consistent cooperation among critically ill patients.

Our focus on the timing of the examination during the course of critical illness adds to the current knowledge about the feasibility of including MMT as part of a research or clinical protocol for patients in the acute phase of critical illness. It may be true that, for many patients, identification of ICUAW before awakening may not affect clinical care or outcome, particularly among those who die in the ICU. However, earlier diagnosis may already inform the prognosis for and management of individual patients, such as those requiring prolonged mechanical ventilation or those with central nervous system pathology [22], and will hopefully identify patients likely to benefit from future therapeutics. Also important is that clinical, translational and basic investigation of the incidence, mechanisms, treatments and outcomes of neuromuscular dysfunction will clearly benefit from a more inclusive approach to the identification of ICUAW.

## Conclusions

In conclusion, although MMT is feasible and reliable in the outpatient setting [10,23,24] and in selected critically ill patients without central nervous system dysfunction [6], neither group is at risk for the acute brain dysfunction that affects most patients during critical illness [25] and precludes early participation in volitional testing. For the subset of critically ill patients who can be assessed with MMT, the agreement between observers regarding the diagnosis of ICUAW is good, particularly when performed after ICU discharge. However, as ICU clinicians and researchers, we cannot be satisfied with restricting our assessment of patients for neuromuscular dysfunction to those who can participate in the examination. Since ICU-acquired neuromuscular dysfunction (including critical illness polyneuropathy and myopathy) is likely associated with severity of illness [4] and may even share the same pathogenesis as septic encephalopathy and acute brain dysfunction [26,27], the patients who cannot be assessed are the most likely to be affected by it.

The ability to detect these physical functional abnormalities early in the course of critical illness is crucial to understanding their incidence and biology, improving prognostication, guiding care, and administering and monitoring interventions designed to prevent or limit the development of ICU-acquired neuromuscular dysfunction. As such, we need to continue to develop and test alternative approaches to the diagnosis of ICU-acquired neuromuscular dysfunction, such as electrophysiologic testing [28-30], nonvolitional strength measurement [31], histology [22,32] and ultrasound [33]. We need to continue to perform MMT for all patients who are able to cooperate, and we need to continue the hard work of attempting to identify and minimize reversible causes of delirium and coma in the ICU [20,34,35]. Interventions such as early mobilization of ICU patients [36] are promising, potentially offering treatments that can decrease delirium, increase patients' ability to cooperate with MMT and decrease ICUAW at the same time.

## Key messages

• Recognition of ICUAW is important.

• Although standardized assessment of ICU patients for weakness using MMT has been proposed, little is known about the feasibility or reliability of this approach.

• We found that most ICU patients cannot participate in MMT during acute critical illness or injury.

• Although we could not assess many critically ill patients with MMT in the ICU, feasibility and interobserver agreement improved after patients' transfer to the hospital ward.

• Nonvolitional approaches to neuromuscular assessment may be important to advance early recognition and potential treatment of ICUAW.

## Abbreviations

ICU: intensive care unit; ICUAW: ICU-acquired weakness; MRC: Medical Research Council; MMT: manual muscle testing; IQR: interquartile range; p-neg: proportion of negative agreement; p-pos: proportion of positive agreement.

## Competing interests

The authors declare that they have no competing interests.

## Authors' contributions

CLH conceived of the study. CLH and BKL designed the study and conducted the study procedures. CLH and ESC performed the statistical analyses. CLH and ESC drafted the manuscript. CLH, BKL and ESC read and approved the manuscript.
